# Epidemiological trends, predictive factors, and projection of tooth loss in Germany 1997–2030: part I. missing teeth in adults and seniors

**DOI:** 10.1007/s00784-020-03266-9

**Published:** 2020-11-21

**Authors:** A. Rainer Jordan, H. Stark, I. Nitschke, W. Micheelis, F. Schwendicke

**Affiliations:** 1 Institute of German Dentists (IDZ), Universitaetsstraße 73, D-50931 Cologne, Germany; 2 Department of Prosthodontics, Preclinical Education, and Dental Materials Science, Rhenish Friedrich Wilhelms University of Bonn, Bonn, Germany; 3 Clinic for Geriatric and Special Care Dentistry, Center of Dental Medicine, University of Zurich, Zurich, Switzerland; 4 Department of Operative and Preventive Dentistry, Charité – Universitätsmedizin Berlin, Berlin, Germany

**Keywords:** Dental caries, Dentistry, Epidemiology, Periodontitis, Tooth loss

## Abstract

**Objective:**

This is the first part of a report on tooth loss in Germany 1997–2030. Here, we describe trends in the prevalence of tooth loss in adults and seniors 1997–2014, assess predictive factors for tooth loss and projected it into 2030.

**Material and methods:**

Data of the cross-sectional, multi-center, nationally representative German Oral Health Studies of 1997, 2005, and 2014 were used. Age, sex, educational level, smoking status, and the cohort were used for ordinary least square regression to assess the association of predictors with tooth loss (missing teeth, MT). The yielded regression coefficients were used to predict tooth loss in 2030.

**Results:**

Compared with 1997, the mean MT in adults (35–44 years old) in 2030 was predicted to decrease by two-thirds to 1.3. The prevalence of tooth loss (MT > 0) will decrease by 72% from 1997 to 2030. In 2030, half of the population of adults will not exhibit any tooth loss. Compared with 1997, the mean MT among seniors (65–74 years old) will decline to 5.6 teeth (i. e. two-thirds reduction) until 2030. Prevalence of tooth loss will be halved by 2030, and approximately one-third of this age group will not exhibit any tooth loss.

**Conclusions:**

Based on the model used, the trend of a robust decline in tooth loss will become more dynamic by the year 2030. As a result, every second adult will have experienced no tooth loss at all in 2030, and seniors will possess more teeth than they have previously lost.

**Clinical relevance:**

This study presents the trends of tooth loss in Germany for a period of three decades. It provides clinically relevant data for health care planning by 2030.

## Introduction

Dental caries and periodontal diseases are the most common oral diseases and the main cause of tooth loss [1]. From an epidemiological point of view, caries experience has been continuously declining since the 1990s in Germany, both in children, adolescents, and among adults, which has a subsequent impact on the rate of tooth loss, especially in the first half of life [2]. The number of periodontally affected teeth, however, has been increasing in parallel [3], with the majority of these teeth being retained long term, also into higher age. Concomitantly, the older population segment is growing, while younger groups are shrinking. Overall, there is a strong indication for oral health gains in a large segment of the life curve on an individual level, but a compression of morbidity in elderly individuals and also older population segments [4].

Tooth loss is a very robust oral-epidemiological health marker, as it is relatively easy to measure. It is highly relevant to patients. Tooth loss is thus an epidemiological key variable and is also used to assess the quality of a dental healthcare [5]. Tooth loss exceeding a certain number of teeth has a significant impact on chewing function, nutrition, speech, and esthetics. This is even more true for complete tooth loss (edentulism), which is the final event of tooth loss and has a specific impact on both quality of life and therapy options. Edentulism is, at least in many high-income countries, a phenomenon of the older population [6–8].

There are only a few longitudinal studies that can identify trends in tooth loss and associate them with trends in caries experience and prevalence, or extent and severity of periodontal disease [9–12]. Cross-sectional surveys may be used as an alternative for identifying trends, but require the consideration of possible cohort effects. Such cross-sectional studies are frequently used for epidemiological population monitoring and for deducing interventions in healthcare planning.

**Fig. 1:**
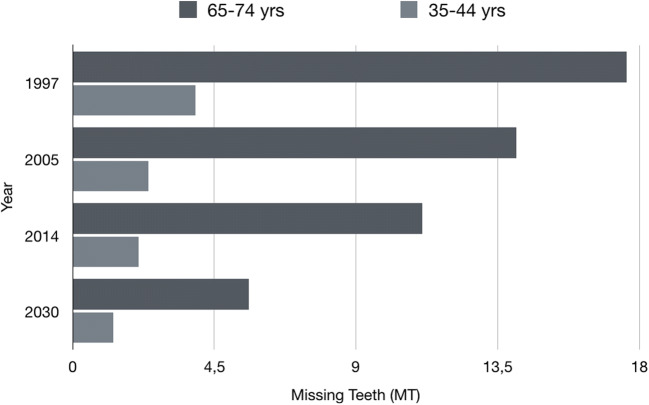
Mean missing teeth (MT) in different age cohorts and waves of the German Oral Health Studies (DMS)

The present study is the first part of a report on tooth loss in Germany. The aim of this study was [1] to illustrate the past trends in tooth loss and edentulism in Germany in the past quarter century on the basis of data from three waves of German oral health studies, [2] to evaluate factors that can predict tooth loss and edentulism at the population level, and [3] to use these predictors to forecast tooth loss and edentulism in the year 2030. This first report is devoted to tooth loss in particular. This is clinically relevant for all adult age groups. In a second paper, we report on edentulism in more detail, focusing on seniors (Fig. 1).

## Materials and methods

The Transparent Reporting of a multivariable prediction model for Individual Prognosis Or Diagnosis (TRIPOD) initiative developed a transparent set of suggestions for the reporting of studies developing, validating, or updating a prediction model, whether for diagnostic or prognostic purposes. The reporting of this study (part 1 and part 2) follows the TRIPOD statement [10].

### Source of data

This study involved pseudonymous data from three waves of the German Oral Health Studies (Deutsche Mundgesundheitsstudien, DMS): DMS III from 1997 [13], DMS IV from 2005 [14], and DMS V from 2014 [15, 16]. The studies were conducted by the Institute of German Dentists (Institut der Deutschen Zahnärzte, IDZ). DMS are cross-sectional, multi-center, nationally representative, clinical and socio-epidemiological studies to investigate the oral health status and behavior of the German resident population in several age cohorts.

### Participants

Study participants were drawn from local residents’ registration offices out of 90 randomly selected communities. A disproportional sample point selection was used, resulting in 60 study sample points in the Western federal states of Germany and 30 study sample points in the Eastern states with post hoc re-dressing. Stratification was performed according to federal states and levels of urbanization. Our data analysis accounted for this complex sample and the associated weighting (see below). The names and addresses of the study participants to be invited were drawn from the registration files of the local residents’ registration offices. Participants were drawn from three age groups: 12 years old, 35 to 44 years old, and 65 to 74 years old; and, only in 2014, 75 to 100 years old.

### Sample size

Per DMS wave, it was the aim to include 1000 subjects (net) per each age group into the study. To achieve this, 2000 participants were sampled per age group. For the DMS III, 3065 participants were included (response rate of 63.6%); for DMS IV and V, these numbers were 4631 (63.1%) and 4609 (50.1%), respectively. Empirical non-responder analyses were conducted to compare the socio-dental characteristics of responders with the target population according to gender, educational level, dental visiting patterns, and dental/prosthodontic status. Non-response bias was found to be minimal.

Here, only the age groups of 35 to 44 years old (younger adults) and of 65 to 74 years old (younger seniors) are reported. This was done, as the older senior group was not available in 1997 and 2005, and as tooth loss was virtually absent in the 12-year-old age group (2.1% in DMS III, 1997; 1.0% in DMS IV, 2005; and 1.2% in DMS V, 2014). Overall, 2546 younger adults (DMS III 1997, 655; DMS IV 2005, 925; DMS V 2014, 966) and 3449 younger seniors (DMS III 1997, 1367; DMS IV 2005, 1040; DMS V 2014, 1042) were included in this study.

### Dental examinations and outcomes

The socio-scientific survey and the oral examination were carried out at venues of the local health authorities. A paper questionnaire was completed by the subjects before clinical examination. The following clinical parameters were assessed: tooth loss, dental caries, periodontal disease, prosthodontic status, developmental and acquired dental hard tissue, and mucosal lesions. To ensure reproducibility, the dental investigators were trained and calibrated by experts. Multiple reliability checks were performed throughout the field phases.

For the present study, the outcome was tooth loss, expressed as extent (number of missing teeth, MT primary outcome) and prevalence (%MT > 0 secondary outcome). Third molars were excluded for this analysis. Inter-rater reliability (Kendall’s tau) for MT between study dentist examiners as compared to a master dentist was 0.99 (DMS III, 1997), 0.89 (DMS IV, 2005), and 0.95 (DMS V, 2014). Data on edentulism can be found in the second part of this paper.

### Predictor variables

Predictor variables were recorded at the beginning of the participants’ examination using self-administered validated questionnaires. Note that for the present study, only those predictors that were concurrently available in socio-demographic projections for 2030 were used. The following predictor variables were used: (1) age in years; (2) sex; (3) educational level as low, middle, or high; (4) smoking status as never, former, and current; and (5) the cohort (coded via study wave as dummy variable DMS III, 1997; DMS IV, 2005; DMS V, 2014).

### Missing data

Missing predictor variables occurred very rarely (< 2% of cases, < 1% of entries) and were handled as missing at random, with exclusion of the respective subject from the model. Multiple imputation using the simple random imputation method was performed in a sensitivity analysis but yielded very similar coefficients allowing this procedure.

### Statistical analysis

Age cohort regression models were created to examine the association between predictor variables and the primary outcome (MT), and to deduce future trends [17]. Ordinary least square linear regression models were used for this purpose. All predictors were entered jointly (only multivariable models were used). A number of interaction terms were tested, without the model being more appropriate. Regression coefficients, expressing the mean difference in MT, and 95% confidence intervals (CI), were used to present the risk of tooth loss. Differences were considered to be statistically significant at *p* < 0.05. Statistical analysis was performed using SPSS 22 (IBM, Armonk, USA) plugged into the open-source software R (version 3.1, the R foundation).

### Model validation

Model validation was performed via split-sample validation comprising two random half of each cohort (stratified by age group). The coefficients yielded from the model developed in one half were used to predict the number of lost teeth in the other half.

### Projection 2030

To make projections of MT in 2030, the yielded regression coefficients from the validated models were used and applied them to the predicted population of younger adults and younger seniors in 2030. These population age ranges were chosen as tooth loss was expected to occur in relevant numbers from adults onwards. To do so, predictor data were collected from a number of sources:

1. Predicted demographic data were drawn from the national statistical office [18].
2. Sex proportions in these age groups were estimated for 35 to 44 years old at 48% female and 52% male in 2030, and for 65 to 74 years old exactly the other way round; 52% female and 48% male [18].
3. Socio-educational status was assumed to be carried forward from the population 35–44 years in 1997, as educational status to change greatly after that age was not assumed, with 30%, 41%, and 29% of individuals being in the low, medium, and high social group, respectively. For the future younger adults, the socio-educational status to the same as in 2014 was assumed.
4. Smoking status was derived from predictions made by the WHO, with stratification for sex [19]. Thirty-two percent of current smokers were assumed, 19% former smokers in the younger adult group, and 29% current and 19% former smokers in the younger senior group.

Spreadsheet-based Monte Carlo simulations were used to predict MT for a simulated population of 3000 individuals (as could be expected to be drawn by the 2030 wave of the DMS 7). Parameter uncertainty was introduced by randomly sampling variables from a binomial distribution. Each population was modelled 100 times, yielding mean MT and 95% CI. YASAIw (University of Washington) plugged into Microsoft Excel (Microsoft, Redwood, USA) was used for this modeling.

Population level estimates were calculated using past and predicted demographic data from the national bureau of statistics [18] with absolute and relative differences being calculated over the 33-year period (1997–2030). The population level estimates were further divided into different sex and socio-educational strata as well as according to smoking status.

## Results

The overall characteristics of the sampled cohorts in 1997, 2005, and 2014 are displayed in Table 1. While sex proportions remained relatively stable in both age groups over time, education level increased. Smoking decreased mainly in younger adults, but not in younger seniors.

**Table 1:** Characteristics of included subjects in different age cohorts and waves of the German Oral Health Studies (DMS). **a** 35 to 44 years old, **b** 65 to 74 years old

Parameter		DMS III (1997)		DMS IV (2005)		DMS V (2014)	
		*n*	%	*n*	**%**	*n*	%
(a) 35 to 44 years old							
Total age cohort		655	100	925	100	966	100
Age (years)	Mean (SD), range	39 (3)	35–44 y	39 (3)	35–44 y	40 (3)	35–44 y
Sex	Male	332	50.7	471	50.9	485	50.2
	Female	323	49.3	454	49.1	481	49.8
Educational level	Low	195	30.0	222	24.3	265	26.5
	Medium	261	40.3	374	40.9	350	36.3
	High	192	29.6	318	34.8	358	37.2
Smoking pattern	Never	264	40.6	402	43.9	425	44.1
	Former	139	21.4	191	20.9	249	25.9
	Current	247	38.0	332	35.2	289	30.0
Tooth loss	Prevalence (%)	502	76.6	544	58.8	549	56.8
	Mean MT (SD), range	3.9 (2.8), 0–28	0–100	2.4 (2.8), 0–28	0–100	2.1 (2.9), 0–28	0–100
(b) 65 to 74 years old							
Total age cohort		1367		1040		1042	
Age (years)	Mean (SD), range	69 (2.7)	65–74 y	69 (2.7)	65–74 y	70 (2.9)	65–74 y
Sex	Male	578	42.2	480	46.2	489	46.9
	Female	789	57.8	560	53.8	553	53.1
Educational level	Low	1031	75.9	666	65.8	642	63.9
	Medium	183	14.0	183	18.1	190	18.9
	High	145	10.7	163	16.1	173	17.2
Smoking pattern	Never	815	60.1	627	62.0	545	53
	Former	358	26.4	305	29.9	362	34.9
	Current	183	13.5	88	8.6	131	12.6
Tooth loss	Prevalence (%)	1342	98.2	1013	97.4	1004	96.3
	Mean MT (SD), range	17.6 (9.1), 0–28	0–100	14.1 (9.8), 0–28	0–100	11.1 (9.1), 0–28	0–100

MT and the prevalence of tooth loss decreased with time in both age groups. While MT decreased by nearly 50% in both age groups between 1997 and 2014, the prevalence of tooth loss decreased by 20% in younger adults, but only by 2% in younger seniors.

All predictors were significantly associated with MT (Table 2); that are the age per life year, sex, educational level, smoking behavior, and the cohort (DMS wave). With each year of age, MT increased in younger adults by 0.195 teeth and in younger seniors by 0.509 teeth. MT was also higher in females than males, those from low versus medium and high educational level, and in current versus former and never smokers. The model was robust when split-sample coefficients were tested in the other half of the sample (the predicted prevalence in the different waves deviated by < 2% when compared to reported prevalence).

**Table 2:** Multivariate analysis. **a** 35 to 44 years old, **b** 65 to 74 years old (log-likelihood **a** 438.826, **b** 790.833; B = regression coefficient)

Parameter	Category	B	95% CI	*P* value
(a) 35 to 44 years old				
Cohort (ref: 1997)	2005	−1.547	−5.321; −1.305	0.001
	2014	−1.758	−2.113; −1.404	< 0.001
Age	Per year	0.195	0.147; 0.243	< 0.001
Gender (ref: male)	Female	0.365	0.084; 0.645	0.011
Educational level (ref: low)	Medium	−1.236	−1.596; −0.875	< 0.001
	High	−2.250	−2.625; −1.875	< 0.001
Smoking (ref: never)	Former	0.186	−0.174; 0.546	0.311
	Current	1.473	1.146; 1.800	<0.001
(b) 65 to 74 years old				
Cohort (ref: 1997)	2005	−3.066	−3.793; −2.340	< 0.001
	2014	−6.028	−6.718; −5.337	< 0.001
Age	Per year	0.509	0.406; 0.612	<0.001
Gender (ref: male)	Female	1.806	1.175; 2.437	< 0.001
Educational level (ref: low)	Medium	−4.020	−4.790; −3.250	< 0.001
	High	−5.717	−6.526; −4.909	< 0.001
Smoking (ref: never)	Former	2.111	1.414; 2.808	< 0.000
	Current	5.403	4.568; 6.349	< 0.001

The yielded coefficients were then applied to project MT and tooth loss prevalence in 2030 (Table 3). The projected MT in younger adults was 1.3 and was 5.6 in younger seniors. Predicted prevalence of tooth loss for the year 2030 in younger adults and younger seniors is 50% and 65%, respectively. In absolute terms, these were 36 and 65 million fewer MT in 2030 compared with 1997, respectively. The reduction in MT was higher in those from a lower than medium and high educational level.

**Table 3:** Population-level estimates of tooth loss in Germany from 1997 to 2030 and relative and absolute changes between 2030 and 1997; **a** 35 to 44 years old, **b** 65 to 74 years old

		DMS III (1997)			DMS IV (2005)			DMS V (2014)			DMS 7 2030			Number of MT change 2030–1997	
Parameter		Population (million)	MT sum (million)	Mean MT	Population (million)	MT sum (million)	Mean MT	Population (million)	MT sum (million)	Mean MT	Population (million)	MT sum (million)	Mean MT	MT diff (million)	relative (%)
(a) 35 to 44 years old															
Total		12.7	49.5	3.9	13.9	33.4	2.4	9.9	20.8	2.1	10.6	13.8	1.3	−36	−72
Sex	Male	6.4	23.7	3.7	7.1	31.1	4.4	5.0	8.9	1.8	5.4	5.4	1.0	−18	−77
	Female	6.3	25.1	4.0	6.8	15.7	2.3	4.9	11.3	2.3	5.2	7.3	1.4	−18	−71
Educational level	Low	3.8	20.6	5.4	3.3	12.7	3.8	2.6	9.0	3.5	2.3	5.4	2.3	−15	−74
	Medium	5.1	18.8	3.7	5.7	14.8	2.6	3.7	7.3	2.0	3.6	5.8	1.6	−13	−69
	High	5.2	13.5	2.6	4.9	4.9	1.0	3.7	4.0	1.1	4.7	4.7	1.0	−9	−66
Smoking pattern	Never	5.2	16.7	3.2	6.1	9.2	1.5	4.5	6.7	1.5	5.2	5.2	1.0	−11	−69
	Former	2.8	8.7	3.1	2.9	6.4	2.2	1.2	2.1	1.7	1.1	1.2	1.1	−7	−86
	Current	4.8	24.1	5.0	4.9	16.5	3.4	3.0	9.5	3.2	3.2	5.1	1.6	−19	−79
(b) 65 to 74 years old															
Total		7.4	130.2	17.6	9.2	129.7	14.1	8.4	93.2	11.1	11.6	65.0	5.6	−65	−50
Sex	Male	3.2	52.5	16.4	4.3	56.8	13.2	3.9	42.9	11.0	5.6	30.2	5.4	−22	−42
	Female	4.2	77.3	18.4	4.9	77.9	15.9	4.5	50.4	11.2	6.0	46.8	7.8	−30	−39
Educational level	Low	5.6	103.8	18.7	6.1	66.2	10.9	4.0	51.6	12.8	3.1	18.2	5.8	−86	−82
	Medium	1.0	14.2	14.8	1.3	10.6	8.4	2.2	20.0	9.0	4.2	20.9	5.0	−7	−47
	High	0.9	11.5	13.5	1.6	21.1	13.5	2.2	16.0	7.4	4.3	18.9	4.4	−7	−64
Smoking pattern	Never	4.7	79.7	17.1	5.7	77.6	13.6	4.7	44.7	9.5	7.1	37.5	5.3	−42	−53
	Former	2.1	35.2	17.0	2.9	49.1	17.2	2.8	33.3	12.0	3.5	23.0	6.6	−12	−35
	Current	0.7	15.5	21.0	0.6	7.7	12.0	0.9	14.4	15.6	1.0	8.2	7.9	−7	−47

Looking at tooth loss in relation to the dental arch, it is noticeable that the general decrease in tooth loss over time has occurred equally in both age groups and in anterior and posterior teeth, respectively. Interestingly, the anterior tooth loss in younger seniors is more pronounced than in the posterior area, whereas the distribution in younger adults is almost symmetrical (Appendix Table 4).

## Discussion

This study describes trends in tooth loss in adults and younger seniors in Germany as measured by repeated waves of a population-representative cross-sectional survey. In our sample and based on nearly 6000 participants, the strongest predictors for MT (and edentulism, see part II) were the cohort, patients’ age, and education level as well as smoking status. In this respect, our findings are in line with previous evaluations of tooth loss [20, 21].

In addition to the known risk factors that can lead to tooth loss, so-called dental generations can also be identified which, as a result of cohort effects, orally represent the current state of health technology in dental care. Our findings regarding the different “dental generations” [22] demonstrate the association between the caries decline and tooth loss. The adults sampled into DMS IV and DMS V (2005, 2014) have benefitted from the wide availability of fluoride toothpaste and preventive programs (fluoride varnish and sealant application in dental practice and group prophylaxis in kindergartens and schools) installed in the late 1970s and 1980s, respectively. In contrast, the adults sampled into DMS III (1997) and the seniors of DMS V had experienced higher caries increments and had been mainly treated restoratively, eventually leading to the final endpoint of the disease, tooth loss. The seniors from DMS III and IV had even entered adulthood with some missing teeth; the extent then increased even further up until age 65–74 years. The three cohorts of the DMS thus represent the “denture,” the “filling,” and the “fluoride” generation: given the chronic-cumulative nature of dental diseases, the endpoint tooth loss can be used to characterize them.

Edentulism can be considered a special case of tooth loss, namely the state after the final “target event.” Despite the closeness of tooth loss and edentulism, there exist clear epidemiological distinctions between these two states. For example, their prevalence is highly different; while tooth loss is highly prevalent in both age groups, edentulism is practically nonexistent among adults. Moreover, the changes observed over time (1997–2014) between both states are remarkable: While the prevalence of tooth loss decreased by one quarter in younger adults (from 77 to 57%), it was almost unchanged in younger seniors (from 98 to 96%). This was also reflected in trends on edentulism, which halved (from 22 to 12%) in younger seniors but remained stable (from 1 to 0.8%) in younger adults (see part II of this paper). It is thus relevant to evaluate both states separately, also given the different treatment needs and subjective impact emanating from them.

Our findings can be contrasted with that from other studies. In accordance with our data, one Finnish study reported a significant decrease of MT between 1978 and 1997, together with a decrease in prevalence from 80 to 60%. The number of adults with reduced dentition (MT > 6) decreased from 26 to 14% with time [23, 24]. A Swedish study reported that between 1975 and 1997, the number of adults with tooth loss decreased from 23 to 10%, whereas this number remained almost constantly among younger seniors (at 25%) [25]. Globally, severe tooth loss in the permanent dentition has been nearly halved since 1990 from almost 40 to 20%, again in line with our data [26].

This study has a number of strengths and limitations. One strength is that the available data are highly intrinsically consistent, as all three examinations were developed, collected, and evaluated by the same study center. Second, the used analytic method for the projection had been tested, validated, and applied to project edentulism, too (see part II); the present study supplements this method from a binary to a continuous outcome and helps to describe the oral health status in Germany in more depth. As limitation, it was not possible to draw testable, causal connections from our analysis given the (repeated) cross-sectional character of the underlying data. Thus, no analysis as to the reasons of tooth loss and the associated pathways, involving dental caries and periodontitis, was possible. Rather, this is a descriptive analysis and projection. The future wave of the DMS in 2021 will, for the first time, allow re-examining the participants of the DMS V and will thus be the first population-representative longitudinal oral health observation in Germany. Second, only a limited number of predictor variables were used in our study, mainly dictated by the availability of predictions of these variables in 2030, whereby the percentage of explained variance (R-squared) fluctuates, depending on the incorporated DMS waves, between 7.8% and 13.5% for the adult group, and between 7.7% and 8.9% for the senior group. These R^2^ values thus appear moderately strong at best. While it was thus not able to reliably predict the individual tooth loss, the findings (from the validation) clearly demonstrate the ability to predict tooth loss on population level. In any case, our projection is only as robust as the predictions of these variables are, and prone to unforeseen social or demographic but also economic etc. changes. Third, our model was validated only in the population it was developed in using the split-sample technique; it will most likely be less accurate when applied to other populations. Thus, a different morbidity dynamic with regard to tooth loss in Germany could emerge in the medium term if global migration continues and, in addition, Germany will compensate for the lack of skilled workers in the future through a targeted immigration policy. An epidemiological study of oral health and access of older migrants in Germany has shown that the number of missing teeth of migrants is only insignificantly higher than that of non-migrants of the same age (70.2 yrs. of age) (MT migrants 14.4 vs. MT non-migrants 12.6; *p* = 0.301) (27). However, as this is a first orienting study, it is not possible to assess with certainty how tooth loss will develop in Germany in the future in times of global migration and immigration. Fourth, our primary outcome was the extent of tooth loss (MT), not the prevalence. The model for the prediction of prevalence was not validated; the projected prevalence should thus be regarded with caution. Last, only two selected age groups were analyzed. Our absolute estimates are not able to catch the burden of tooth loss in the whole German population. Given the expected demographic shift and the only recently measured oral health status of older seniors (in the DMS V), assessing and projecting tooth loss in the very old seems highly relevant, too, and should be performed in future studies. Especially in this age group, linking tooth loss data with general health or further socio-demographic parameters is of interest, also to gauge causal (bi-directional) pathways and to determine the subjective impact of (also non-severe) tooth loss (and tooth replacement). The Study of Health in Pomerania (SHiP), for example, links tooth loss and further general health data [27]. Eventually, a further triangulation with qualitative sociological research might also assist a deeper interpretation of tooth loss findings.

## Conclusion

In conclusion, and within the limitations of this study, a robust and continuous decrease in tooth loss in the German population has occurred between 1997 and 2014. Based on our projection, this trend will become more dynamic by the year 2030, with around 50% of adults not having experienced any tooth loss in 2030, while young seniors will possess more teeth than they have previously lost. This decrease is likely to reduce the need for tooth replacement treatments, while the challenges of retaining more teeth for longer (for example, with regard to periodontal diseases and root caries) will need to be actively addressed.

## Appendix

**Table 4:** Population-level distribution of tooth loss (MT) in Germany from 1997 to 2014; (a) 35- to 44-year-olds, (b) 65- to 74-year-olds.

		Mean MT	Prevalence (%)							
			MT = 0	MT = 1	MT = 2	MT = 3	MT = 4	MT = 5	MT = 6	MT = 7
(a) 35 to 44 years old										
Anterior teeth	DMS III (1997)	2.2	40.5	18.2	12.5	6.6	8.7	3.8	1.2	1.4
	DMS IV (2005)	1.4	52.8	17.5	12.0	6.4	5.0	1.8	1.0	0.6
	DMS V (2014)	1.0	62.8	14.2	10.2	3.9	5.9	.9	.6	0
Posterior teeth	DMS III (1997)	2.13	29.0	19.5	16.9	13.0	9.8	4.3	3.1	2.4
	DMS IV (2005	1.46	43.4	18.9	14.5	10.2	5.7	3.0	1.5	1.2
	DMS V (2014)	.93	59.1	16.1	11.6	6.0	4.0	1.0	.7	.4
(b) 65 to 74 years old										
Anterior teeth	DMS III (1997)	11.7	4.7	5.3	4.6	5.1	5.0	3.0	3.7	3.7
	DMS IV (2005)	9.0	9.9	10.1	8.5	6.4	5.7	4.9	3.5	3.6
	DMS V (2014)	6.3	16.7	12.6	12.8	8.7	6.7	4.8	3.9	3.6
Posterior teeth	DMS III (1997)	6.4	2.3	2.9	2.9	4.5	7.4	8.6	9.9	10.9
	DMS IV (2005)	5.4	5.1	5.5	8.0	8.8	8.1	9.8	1.0	8.1
	DMS V (2014)	4.4	8.4	9.8	8.9	11.2	12.7	11.5	8.6	8.0
